# Effects of the Distribution of Female Primates on the Number of Males

**DOI:** 10.1371/journal.pone.0019853

**Published:** 2011-05-16

**Authors:** Laurel Mariah Carnes, Charles L. Nunn, Rebecca J. Lewis

**Affiliations:** 1 The Law School, University of Chicago, Chicago, Illinois, United States of America; 2 Department of Human Evolutionary Biology, Peabody Museum, Harvard University, Cambridge, Massachusetts, United States of America; 3 Department of Anthropology, University of Texas at Austin, Austin, Texas, United States of America; University of Manitoba, Canada

## Abstract

The spatiotemporal distribution of females is thought to drive variation in mating systems, and hence plays a central role in understanding animal behavior, ecology and evolution. Previous research has focused on investigating the links between female spatiotemporal distribution and the number of males in haplorhine primates. However, important questions remain concerning the importance of spatial cohesion, the generality of the pattern across haplorhine and strepsirrhine primates, and the consistency of previous findings given phylogenetic uncertainty. To address these issues, we examined how the spatiotemporal distribution of females influences the number of males in primate groups using an expanded comparative dataset and recent advances in Bayesian phylogenetic and statistical methods. Specifically, we investigated the effect of female distributional factors (female number, spatial cohesion, estrous synchrony, breeding season duration and breeding seasonality) on the number of males in primate groups. Using Bayesian approaches to control for uncertainty in phylogeny and the model of trait evolution, we found that the number of females exerted a strong influence on the number of males in primate groups. In a multiple regression model that controlled for female number, we found support for temporal effects, particularly involving female estrous synchrony: the number of males increases when females are more synchronously receptive. Similarly, the number of males increases in species with shorter birth seasons, suggesting that greater breeding seasonality makes defense of females more difficult for male primates. When comparing primate suborders, we found only weak evidence for differences in traits between haplorhines and strepsirrhines, and including suborder in the statistical models did not affect our conclusions or give compelling evidence for different effects in haplorhines and strepsirrhines. Collectively, these results demonstrate that male monopolization is driven primarily by the number of females in groups, and secondarily by synchrony of female reproduction within groups.

## Introduction

A fundamental aim of behavioral ecology is to understand the factors that influence variation in mating systems across species [1], [2]. A common approach to this question is to investigate the factors that influence the spatiotemporal distribution of males and females, particularly in light of reproductive investment asymmetries [3], [4]. More specifically, the reproduction of males is expected to be limited by access to receptive females [3]–[5], whereas the reproduction of females is expected to be limited by access to resources [6], [7], particularly in mammals. The ability of males to monopolize access to females, and hence their spatial distribution, depends on the distribution of females in space and time [1], [2], [8]–[10]. Thus, variation in the distribution of females is an important evolutionary determinant of reproductive strategies and a crucial explanatory factor with respect to mating and social system diversity [11].

When investigating the factors that influence the number of males in social groups, researchers have considered two major types of factors: *spatial effects* related to the spatial distribution of females in groups of different size and stability, and *temporal effects* related to the distribution of fertile females in a group through time (i.e., patterns of reproductive synchrony). In terms of spatial effects, males may have a reduced ability to exclude other males from reproductive opportunities as female group size increases and/or females become more spatially dispersed [12]. Decreased visibility of females is one reason why dispersion may reduce male reproductive control [10], [13]. In terms of temporal effects, male monopolization potential is also expected to decrease with increased reproductive synchrony among females (i.e., as female temporal overlap increases) because reproductive synchrony may make excluding rivals from reproductive opportunities more difficult [10], [14], [15]. Reproductive synchrony has been measured in several ways, including reproductive seasonality [10], [16], [17], expected female estrous overlap based on demographic and life history parameters [10], [18], [19], and estimates of actual female estrous overlap [10]. Studies of the ability of males to monopolize or control access to fertile females have used proxies such as the operational sex ratio [1], [20], [21], the number of males [10], [16], reproductive skew [22]–[29], and the number of mates [30], [31].

Many studies across a wide range of organisms have documented support for the assertion that male monopolization ability is shaped by the temporal and spatial distribution of females [8], [9], [14], [15], [32]–[44]. However, contrary results have been found. For example, males sometimes distribute themselves based on female density (e.g., field voles, *Microtus agresti*: [45]) or general resource abundance and distribution (e.g., Gunnison's prairie dogs, *Cynomys gunnisoni*: [46]) not mating strategies, and female distribution sometimes is determined by access to males (e.g., white rhinoceros, *Ceratotherium simum simum*: [47]), or to reduce male harassment (e.g., South American sea lions, *Otaria flavescens*: [48]).

Primates exhibit extensive diversity in social and mating systems [7], [49]–[54], and abundant, high quality data are available on group composition, breeding seasonality, life history, and sexual behavior needed for addressing questions related to male monopolization of females [55], [56]. Many researchers have sought to understand the factors that influence the number of males in primate groups [10], [16], [17], [57]–[61] and the distribution of reproduction among males within those groups [62]–[67]. An underlying goal of these studies was to understand the ways in which male primates monopolize reproduction. For example, Mitani et al. [16] and Nunn [10] found that the number of males in a social group increases as the number of females increases, suggesting that primate males have greater difficulty monopolizing access to a larger group of females. Similarly, Kutsukake and Nunn [68] demonstrated that reproductive skew decreases as the number of males in multi-male groups increases, indicating that the presence of more males generally decreases an individual male's ability to monopolize females.

Since Nunn's [10] analysis of the number of males in primate groups, substantial changes in concepts and methodology have occurred and new data have become available, particularly for previously under-studied primates such as lemurs. We focus here on three major questions that have arisen since the publication of Nunn [10]. First, a shift has occurred in the conception of primate sociality [69], [70]. Recognizing the diversity of social systems, grouping patterns are now categorized as cohesive and dispersed based upon the inter-individual distances of social group members. Additionally, fission-fusion societies are recognized to exhibit a continuum of spatial and temporal cohesion, and with this change more species are expected to be identified as having flexible grouping patterns [71]. The spatial cohesion of females may also be important in accounting for variation in the number of males, yet previous studies used only female number as a measure of spatial monopolizability. The distinctions in spatiotemporal cohesion may be particularly relevant in understanding the social organization of many strepsirrhine [72] and hominoid [71] primates because a number of these species are now considered to have dispersed rather than solitary social systems.

Second, previous comparative studies utilized a relatively limited sample of primates. Indeed, the Nunn [10] dataset included only a single strepsirrhine primate (*Lemur catta*). Over 60% of the species in the dataset were cercopithecoids, a taxon that exhibits a number of derived social traits [73]. In the last decade, information on a wide variety of primates, especially lemurs, has increased substantially. An analysis that represents the vast diversity of sociality exhibited in primates is needed to assess the validity of the initial studies. Additionally, an updated analysis will allow for a more rigorous test of the assertion that social systems in lemurs are fundamentally different from anthropoids [13], [74].

Finally, advances in statistical methods and phylogenetic controls have changed how researchers conduct comparative studies. Previous comparative research was conditioned on only one or a few phylogenetic hypotheses. Importantly, in Nunn [10], some results varied depending on whether phylogeny was taken into account and, if so, on the tree that was used. New approaches based on Bayesian statistical methods provide a way to run analyses across a set of trees sampled in proportion to their posterior probabilities [75], [76], and to run these analyses in a way that incorporates the degree of phylogenetic signal and uncertainty in the underlying evolutionary and statistical models [77]–[79]. Thus, rather than using a handful of trees and comparing phylogenetic and non-phylogenetic analyses, the analysis now can be run across phylogenies that are sampled in proportion to their posterior probability, and the degree of phylogenetic signal across these trees can be estimated.

Here, we use a significantly expanded comparative dataset, a recent Bayesian inference of primate phylogeny, and the latest advances in phylogenetic methods to investigate how the spatiotemporal distribution of females influences the number of males in primate groups. Specifically, we hypothesized that the ability of males to monopolize access to females is affected by both the spatial distribution of females and the temporal distribution of reproductive opportunities (i.e., the patchiness of females in space and time). If the spatial distribution of females is important in accounting for the number of males in primate groups, then the number of males is expected to covary positively with (i) the number of females in a group and/or (ii) reduced spatial cohesion of females, measured as greater fission-fusion sociality or dispersed females. If the temporal distribution of reproductive opportunities affects the number of males in primate groups, then we predicted that the number of males in a group (iii) increases with increasing estrous overlap [10]. Such an effect is expected because monopolizing access to females when they are simultaneously receptive should be more difficult for a single male. For similar reasons, we predicted that the number of males increases in lineages characterized by (iv) greater indices of reproductive seasonality and (v) shorter breeding season durations (i.e., a negative association with the number of males). In such situations, females are expected to come into estrous more simultaneously, making it harder for a single male to monopolize reproductive access. In addition, we tested for trait differences in the two major primate suborders (i.e., haplorhine and strepsirrhine primates).

## Methods

Male monopolization ability was assessed as the number of males in primate groups (defined as the number of males in a foraging group). Males are assumed to prefer to monopolize females when possible [80]. The Mitani et al. [16] dataset was supplemented with data on many additional species from the published literature (Table S1). When data for multiple groups were presented in a single source, the numbers were averaged. When data from multiple studies conflicted, preference was given to long-term studies. While this method does not account for intraspecific variation [81]–[83], it is expected to increase our ability to discern evolutionary patterns, it is a common approach in comparative analyses, and it is consistent with our goal of increasing the taxonomic scope of research on the number of males in primate groups.

### The Spatial Distribution of Female Primates

Following previous researchers [10], [16], [18], [55], [58], we used the number of adult females in a foraging group to examine how the spatial distribution of female primates affects the number of males, which we take as a measure of male monopolization potential. In addition, we examined the spatial cohesion of primate groups. Data on female number were compiled as described above for male number. All 71 species (n_strepsirrhine_ = 22, n_haplorhine_ = 49) were also classified as exhibiting cohesive, dispersed, or fission-fusion grouping patterns based on the spatial distribution of females in social groups. A cohesive social group was defined as one in which all group members travel together, forage together, and regularly participate in physical interactions during times of both activity and rest [31]. A dispersed social group was defined as one in which individuals forage primarily solitarily [72]. These individuals may commonly sleep socially or exhibit other social interactions outside of the mating season [72]. Using the Aureli et al. [71] description of fission-fusion dynamics and data from Campbell et al. [55], species exhibiting variable cohesion and party size (including multilevel societies) groups were classified as fission-fusion.

### The Temporal Distribution of Reproductive Opportunities

For measures of the temporal distribution of females, we used (1) data on estimates of estrous overlap based on demographic and life history parameters and (2) two proxies for expected overlap based on breeding seasonality. Expected estrous overlap was calculated following Nunn's [10] modification of the approach originally developed by Dunbar [18], [19]. The level of estrous overlap between females was assumed to be a function of the average number of mating days by individual females per ovulatory cycle, the duration of the mating season, and the number of females in the social group [10]. The probability of *Y* females mating simultaneously was calculated using the binomial expansion [84]:
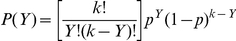
In the above equation, *P(Y)* is the probability of *Y* females mating simultaneously, *k* is the number of females rounded to the nearest integer, and *p* is the probability an individual female is mating. The variable *p* is calculated as two times the quantity of the duration of mating divided by the length of the mating season. The above binomial theorem was used to calculate the probabilities of zero or one female mating simultaneously, and this value was then subtracted from one to give the probability of two or more females mating simultaneously [10]. The probability of co-cycling females is the expected estrous overlap. We did not use the later modification of predicted overlap by Nunn et al. [85] that takes into account the number of cycles to conception and non-fertile matings because high-quality data were not available for coding all the species. Moreover, values from Nunn [10] and Nunn et al. [85] were very highly correlated (r = 0.94, n = 24, 95% credible interval from a Bayesian phylogenetic analysis: 0.93 to 0.95). Because one of our goals was to examine variation in a wider array of primates, we used the older measure of overlap, which strongly predicts measures of synchrony obtained with the newer measure, enabled us to include more species (n_strepsirrhine_ = 22, n_haplorhine_ = 49), and provided more direct comparison to the analyses in Nunn [10].

Birth season duration was used as a proxy for breeding season duration. Birth season duration values from Mitani et al. [16] were supplemented with additional data from the published literature (Table S1). Specifically, birth season duration was scored as the number of days in which 75% of all births fall, following Ridley [17] and Mitani et al. [16]. Data were available for all 71 species.

We also examined a more sophisticated measure of birth seasonality developed by Janson and Verdolin [86], which is based upon seasonality of births and derived using circular statistics. In circular statistics, months can be plotted as a circle of angles with the complete axis equal to one year (365 days). Every birth is plotted as vector of length one with an angle (*a*) [86]. All individual vectors are then summed producing a vector of length *L* and angle *A*. The length of the vector divided by the total number of observations is *r*. Janson and Verdolin's [86] variable *r* measures how unevenly births are distributed across the year, with a value of 0 indicating a perfectly even distribution of births across months and a value of 1 indicating all births occurred at precisely the same time. Data were available for 41 species, including 7 strepsirrhines.

### Analyses

We tested the predictions using regression models that incorporated phylogenetic information scaled by the degree of phylogenetic signal in the residuals (see below). Analyses for each of the response variables included species in the models only if information was available for each of the predictor variables. All continuous variables were log_10_ transformed, and a value of 1 was added to each vector that contained a value of zero prior to the log transformation. We repeated the analyses with a binary coding of taxonomic affiliation to assess whether differences exist between strepsirrhines and haplorhines [87], and we reran a multiple regression model involving key predictors for spatial and temporal effects in strepsirrhines and haplorhines separately.

We included the number of females and either one variable related to the temporal distribution of females (estrous overlap, breeding season, or seasonality) or the binary measure of female spatial distribution. We thus estimated coefficients for the independent effects of spatial and temporal distributions of females. Previously, Nunn [10] obtained residuals from the regression of estrous synchrony on female number, and used those residuals in the analyses to control for the association between female number and estrous synchrony. In our dataset, however, we found no evidence for strong collinearity among these characters (R^2^ = 0.56 in a statistical model that incorporated phylogeny), and thus the regression model should accurately estimate their independent effects. Nonetheless, for comparison to the previous analysis [10], we also conducted additional analyses using residual estrous overlap.

Our statistical models incorporated phylogeny by representing the error term of the statistical model as a variance-covariance matrix that reflects the phylogenetic relationships among the species [88]. We also estimated the parameter λ, which scales the off-diagonal elements of the variance-covariance matrix (corresponding to internal branches of the phylogeny) and serves as a measure of phylogenetic signal [88]. The parameter λ generally lies between 0 and 1. When λ = 0, this corresponds to a non-phylogenetic tests because all internal branches are set to be 0 (i.e., collapsed), resulting in a “star phylogeny” [89]. Values of λ greater than 0 represent increasing phylogenetic signal, with λ = 1 indicating that the given branch lengths adequately account for variation in the trait under a Brownian motion model of evolution.

Phylogenetic relationships and branch lengths are never known with certainty, and thus results should not be conditioned on a single phylogenetic hypothesis [75], [76]. Here, we used a sample of 100 dated phylogenies from a recent Bayesian inference of primate phylogeny (*10kTrees*, [90]), which can be accessed on the Internet (http://10ktrees.fas.harvard.edu/). We used Version 1 of *10kTrees* because it best matched the set of species in our dataset. By running our analyses across this sample of trees, the results were not conditioned on a particular phylogeny or set of branch lengths. To this tree, we added mountain gorillas (*Gorilla gorilla beringei*) as sister species to the Western lowland gorilla (*G. g. gorilla*) with a split date of 1.25 million years ago (Mya; [91]), and we added *Eulemur macaco macaco* as a sister taxon to *E. m. flavifrons* with a split date of 2.34 Mya (based on Version 2 of *10kTrees*, which included both species; [90]). We included *Saguinus fuscicollis* by renaming it *S. tripartitus*, which was not included in our dataset but is closely related to *S. fuscicollis*.

Statistical models were sampled from a Bayesian posterior probability distribution. For this analysis, we fit regression models using the program BayesTraits [76]. BayesTraits uses Markov Chain Monte Carlo (MCMC) to sample regression coefficients and λ, with a different tree randomly selected in each iteration of the chain. We ran the MCMC chain for 1,050,000 iterations and sampled parameter values every 100 iterations, discarding the first 50,000 iterations as burnin. The models used uniform priors on regression coefficients ranging from −100 to 100. We adjusted the “ratedev” parameter to obtain average acceptance rates between approximately 20 and 40% [76], and we repeated all analyses two times to ensure convergence to the same distribution of parameter estimates.

From these analyses, we obtained 10,000 estimates of the coefficients and λ, which reflect a posterior probability distribution of parameter estimates. We calculated the percentage of samples from the MCMC sample in which a parameter value (e.g., a regression coefficient) was in the predicted direction and report those percentages, along with the mean coefficient and 95% credible intervals for λ. If an independent variable has no effect on the dependent variable, we expect its coefficient will be equally represented as positive or negative (i.e., 50% of samples will support the prediction). Percentages closer to 100% reflect greater support for a prediction. For drawing conclusions, we interpret results with >95% of regression coefficients in the predicted direction as “strongly supportive,” between 90% and <95% as “weakly supportive,” and between 85% and <90% as “possible” support in need of investigation with larger sample sizes. By using both a sample of trees and a sample of regression coefficients, both obtained using MCMC, we control for phylogenetic uncertainty and uncertainty in the underlying statistical and phylogenetic models.

## Results

**Figure 1:**
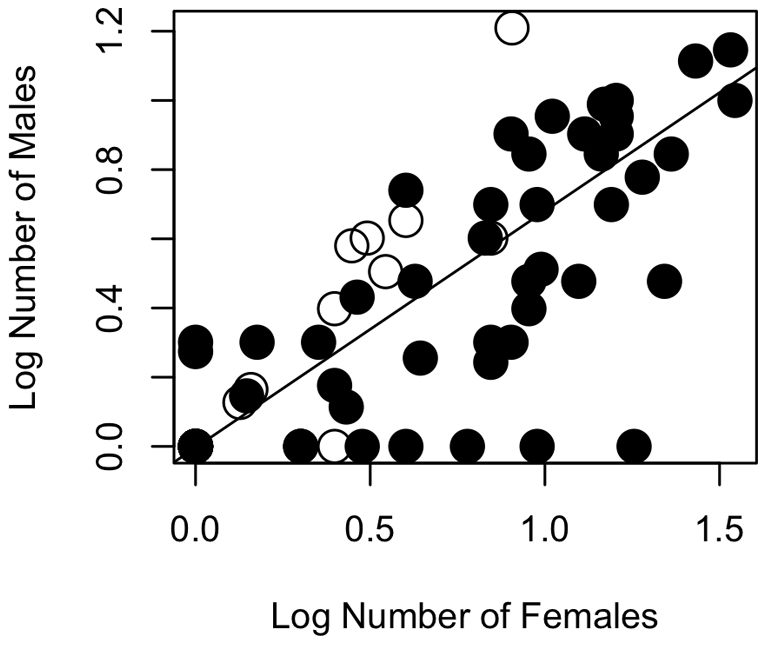
Association between number of males and number of females in primate groups. Data are log_10_ transformed. The regression slope of 0.68 was obtained as the mean of the posterior probability distribution from the MCMC analysis (Table 1, Fig. 2) and was forced on to the raw data. The intercept of −0.0035 was also obtained from the phylogenetic analysis. Haplorhines are represented with black circles and strepsirrhines with open circles.

**Figure 2:**
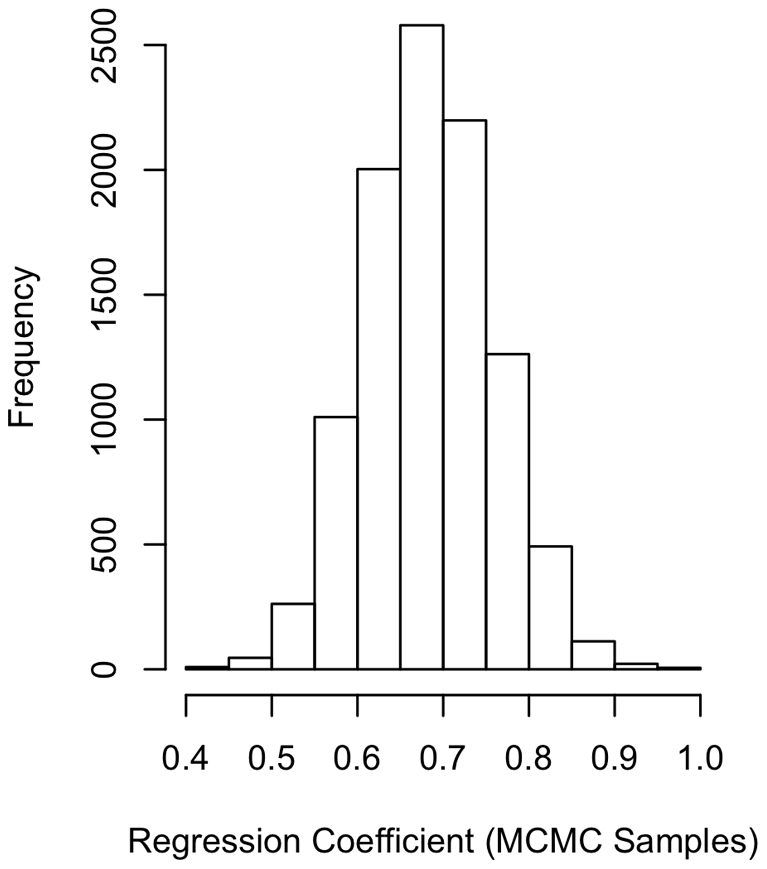
Regression of number of males on number of females. The distribution of regression coefficients was obtained from the MCMC analysis. Note that all coefficients sampled are substantially larger than zero, providing strong support for an association between these two variables.

**Figure 3:**
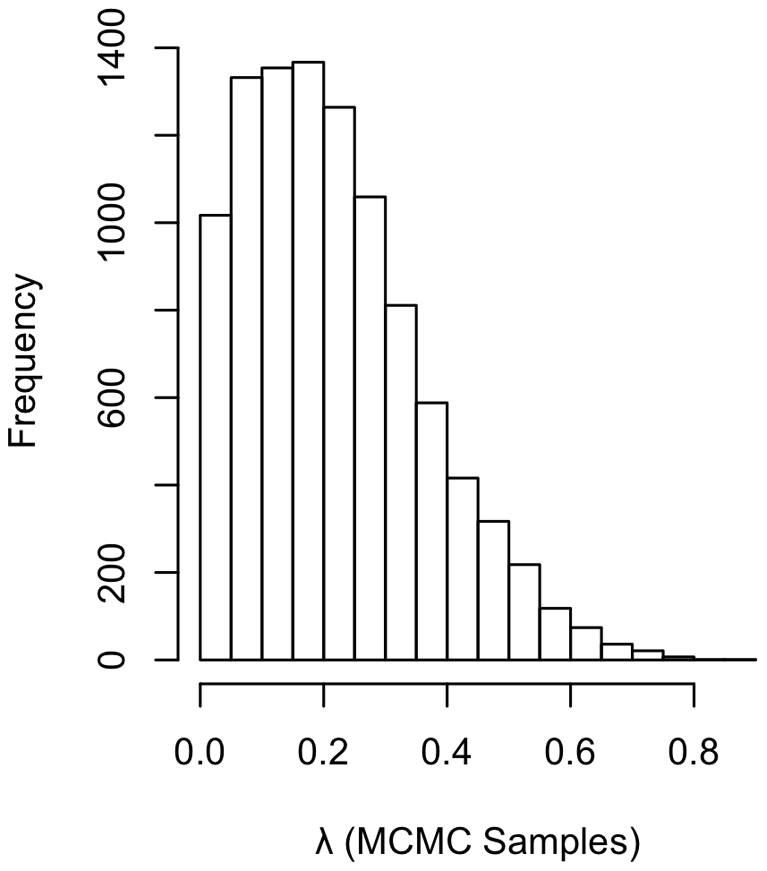
Distribution of λ in regression of male number on female number. The distribution of λ is wide, but shows a peak at about 0.2. Thus, internal branches are scaled to 1/5 of their original lengths. This result suggests low, but meaningful, phylogenetic signal in the data.

In all of our samples across all tests conducted (Table 1), we found that the coefficient relating the number of females to the number of males was positive, in support of Prediction i and the importance of spatial effects (Fig. 1 and 2). Estimates of λ were relatively low (mean of 0.22, Fig. 3), but the 95% credible interval on λ excluded zero. These results suggest that phylogeny has an effect, albeit a weak effect. The methods we used take phylogeny into account according to the degree of signal in the statistical model.

**Table 1:** Predictors of the number of males in primate groups.

Model Statistics			Number of Females			Other Variables			
Prediction	λ Cred. Interval	*R^2^*	Predicted Effect	Mean Coef.	%Support Prediction	Other Variable	Predicted Effect	Mean Coef.	%Support Prediction
(i) # Females	0.01–0.55	0.59	+	0.68	100%	n/a	n/a	n/a	n/a
(ii) Spatial Cohesion	0.02–0.58	0.57	+	0.68	100%	Fission- Fusion	+	−0.01	46.1%
						Dispersed Females	+	−0.04	36.9%
(iii) Estrous Overlap	0.04–0.61	0.61	+	0.52	100%	Estrous Overlap	+	0.17	99.1%
(iv) Breeding Seasonality	0.04–0.81	0.47	+	0.67	100%	Breeding Seasonality	+	0.18	89.7%
(v) Breeding Season Duration	0.01–0.54	0.60	+	0.68	100%	Breeding Season Duration	−	−0.13	93.7%

Notes: The table summarizes statistical tests of the predictions in which the number of males is the dependent variable, and statistical models include the number of females on its own (i) or in combination with two other predictor variables that indicate aspects of female spatial distribution (ii) or in combination with one variable reflecting temporal distribution (iii to v). Predictions follow numbering given in the Introduction. For λ, we give a 95% two-tailed credible interval from the MCMC sample (n = 10,000). *R^2^* is the average value across this same sample (although the range of variation was relatively narrow). For the number of females and other variables, we give the predicted effect, the mean coefficient from the MCMC sample, and the proportion of samples that supported the prediction. If a predictor has no influence on the number of males, we expect only about 50% of the samples to give regression coefficients in the predicted direction.

In a second assessment of spatial effects (Prediction ii), we investigated whether less cohesive groups have more males, as expected if monopolizing individual females in closer proximity to one another is easier for a male. Using dummy variables to identify species as fission-fusion or dispersed, however, we found no evidence in favor of this hypothesis. Thus, in a multiple regression model, the coefficient that reflects fission-fusion sociality's effect on the number of males was positive in only 46% of the samples, and the coefficient for dispersed social systems was positive in only 37% of the samples (as compared to an expected value of 50% for independent variables that have no effect on a dependent variable; Table 1). To determine whether our definitions of dispersed and fission-fusion influenced our results, we reran the analysis with spatial cohesion as a dichotomous variable (cohesive or not) and found similarly non-compelling results (77.4% of the MCMC samples supporting this prediction).

**Figure 4:**
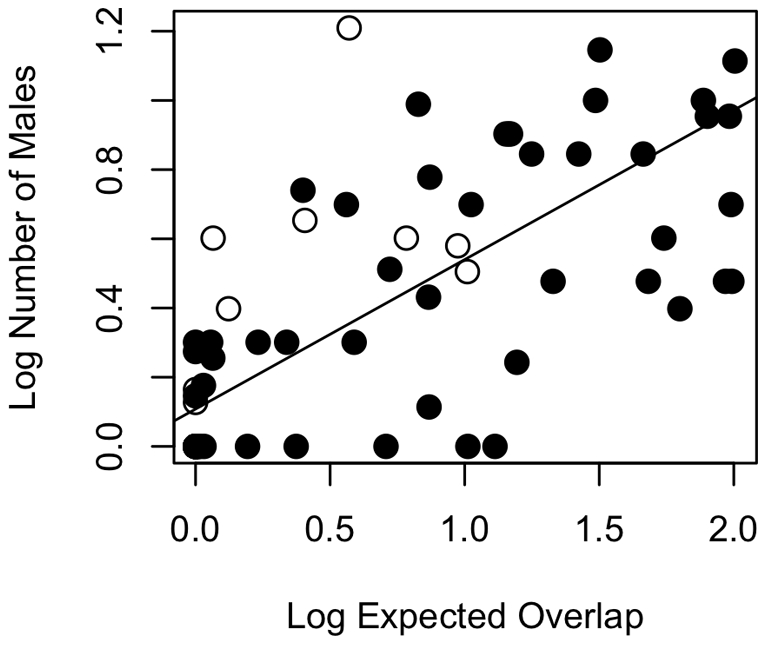
Association between number of males and expected estrous overlap. Data are log_10_ transformed. The regression slope of 0.432 was obtained as the mean of the posterior probability distribution from regressing the number of males on estrous overlap and controlling for phylogeny (R^2^ = 0.47, lambda = 0.29). All 10,000 sampled regression coefficients were positive. The intercept of 0.108 was also obtained from the phylogenetic analysis. Haplorhines are represented with black circles and strepsirrhines with open circles.

**Figure 5:**
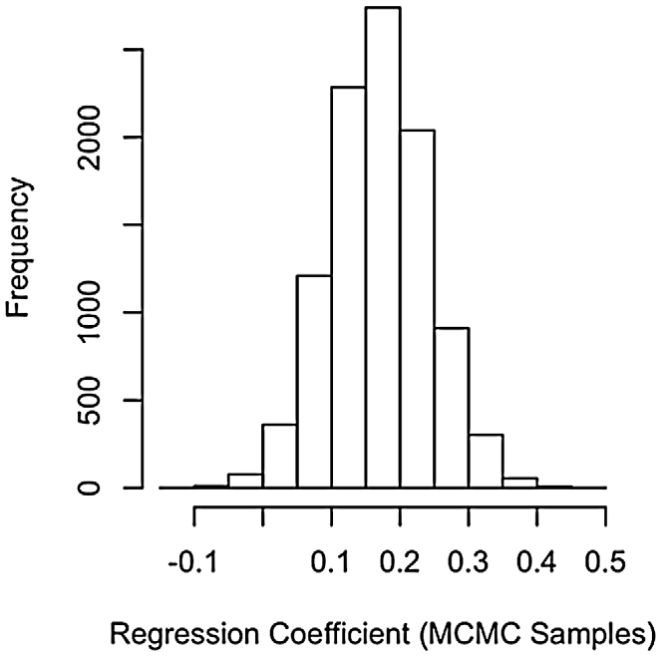
Regression coefficients relating estrous overlap to the number of males. The distribution of regression coefficients was obtained from the MCMC analysis. Note that most coefficients sampled are larger than zero, providing strong support for an association between these two variables.

We also found support for temporal effects, particularly in tests that included estrous overlap as a predictor variable (Prediction iii). This variable was positive, as predicted, in 99.1% of the MCMC samples (Fig. 4 and 5). Thus, independently of the number of females, estrous overlap explains variation in the number of males, as expected if greater overlap makes defending access to a group of females more difficult for a male. Seasonality also showed a tendency to affect the number of males, albeit less so than estrous overlap, with greater seasonality covarying with more males in primate groups in 89.7% of the sampled regression coefficients from the MCMC analysis (Prediction iv). The duration of the breeding season itself appeared to be a better predictor (Prediction v), with 93.7% of the MCMC samples revealing the predicted negative relationship between these two variables. Differences in the strength of findings for Predictions iv and v may be related to sample sizes, with substantially more data available for Prediction v (n = 71) than Prediction iv (n = 41).

Although we found that estrous overlap positively predicted the number of males in primate groups in nearly all of the MCMC samples, including estrous overlap in the statistical model did not account for noticeably more variation (*R^2^*) in the regression model (e.g., compare predictions i and iii in Table 1). The estimated regression coefficient for the number of females became smaller when estrous overlap was included in the model, however. This finding suggests that the number of females, when used on its own in a statistical model, is already capturing some temporal effects; when temporal effects are incorporated more directly, the regression coefficient declines, with temporal effects then captured by the measures of (or proxies for) estrous overlap.

Based on these findings and following Nunn [10], we regressed expected overlap on the number of females (mean slope = 1.06, with 100% of coefficients positive; mean λ = 0.81). We then used the residuals from this model as a measure of estrous overlap that is statistically independent of the number of females. Residual overlap remained a strong predictor of the number of males in the group in a multiple regression model that also included the number of females (99.0% of regression coefficients for residual overlap were positive, mean λ = 0.28).

### Suborder and Female Distributive Traits

Table 2 provides phylogenetically controlled tests of differences among the suborders. The analyses revealed a trend for haplorhines to have more males and females per group, and for strepsirrhines to be more seasonal in their mating. Statistical models with only binary suborder codes gave evidence for phylogenetic signal, but the models explained no more than 2% of the variation in the data, and often substantially less. Thus, suborder is not a key factor that accounts for variation in primate demographic variables, estrous overlap, or breeding seasonality.

**Table 2:** Differences in strepsirrhines and haplorhines.

Variable	%Models Favoring Higher Values in Haplorhines	Mean Coef.	*R^2^*	λ Credible Interval
# Males	83.4%	0.19	0.02	0.02 to 0.63
# Females	83.3%	0.32	0.02	0.23 to 0.87
Expected Overlap	81.0%	0.45	0.01	0.30 to 0.89
Residual Overlap	60.2%	0.13	0.001	0.51 to 0.98
Breeding Seasonality	19.7%	−0.34	0.02	0.40 to 0.96
Breeding Season Duration	64.5%	0.15	0.002	0.81 to 1.00

We also investigated whether including suborder in the statistical models for predictions in Table 1 changed those results. Results remained largely the same, with only weak indications that effects may vary among the suborders in slight ways. In particular, haplorhines show a trend to have fewer males than strepsirrhines, after controlling for the number of females and the degree of expected estrous overlap. Thus, with regard to prediction i, in 79.7% of the MCMC samples, the number of males declines in haplorhines relative to strepsirrhines. Although not strong evidence, it is opposite to what was found when comparing the number of males in the two suborders (Table 2). Similarly, in tests of Prediction iii, 83.4% of sampled regression coefficients predict smaller numbers of males in haplorhines relative to strepsirrhines. In this model, 99.3% of samples show a positive relationship between estrous synchrony and the number of males in primate groups. Similar results were found for the other predictions, with results consistent with findings when suborder was not included in our statistical models.

Finally, we reran analyses for strepsirrhines and haplorhines separately, focusing on a multiple regression model relating the number of males to the number of females and female estrous overlap. For both primate radiations, we found strong effects of both female number and female overlap as predictors of the number of males in primate groups (haplorhines: *b_female_number_*>0 in 99.95% of MCMC samples, *b_female_overlap_*>0 in 98.8% of MCMC samples, R^2^ = 0.57, mean λ = 0.32; strepsirrhines: *b_female_number_*>0 in 100% of MCMC samples, *b_female_overlap_*>0 in 98.3% of MCMC samples, R^2^ = 0.87, mean λ = 0.43).

## Discussion

The spatial distribution of females and the temporal distribution of reproductive opportunities exert a powerful influence upon male reproductive strategies and are key determinants of animal mating and social systems [11]. Using the diversity present in primates, we tested the extent to which male strategies to monopolize reproduction are shaped by the spatiotemporal distribution of females. First, in terms of spatial effects, female group size, but not spatial cohesion, was found to exert a strong influence on the number of males in primate groups in all of our analyses. Second, previous studies of male monopolization potential provide inconsistent support for the hypothesis that the temporal distribution of females affects the number of males in primate groups (Table 3), including a previous study that found different results when using different hypotheses of primate phylogeny [10]. The results of our more expansive study suggest that the temporal distribution of females does affect the number of males in a social group, and consistent with previous findings, this effect is independent but secondary to the number of females in a group.

**Table 3:** Summary of significant results and comparison with significant results of similar studies.

	Mitani et al. (1996)	Nunn (1999)	Lindenfors et al. (2004)	Present Study
# Females	Y	Y	Y	Y
Synchrony	n/a	Y	n/a	Y
Mating Season Duration	N	Y	n/a	Y
Breeding Seasonality	n/a	n/a	n/a	Possible Y
Spatial Cohesion	n/a	n/a	n/a	N

Y = yes, a significant relationship with the number of males is present; N = absence of a relationship with the number of males; n/a = not applicable, the study did not examine this measure of female distribution.

The results of our study significantly extend a previous phylogenetic study of the number of males in primate groups [10] by increasing the taxonomic scope and by using a more sophisticated statistical approach. The number of species examined increased by nearly 50% and substantially increased the representation of a major primate radiation. Knowledge of strepsirrhine behavior and ecology, in particular, has greatly increased over the last decade, permitting a more expansive dataset than previous studies. Given that sex ratios in lemur societies have been reported to differ from anthropoids [13], one might expect that a dataset with 23% lemurs would result in substantially different conclusions than a dataset comprised of only a single lemur species, indeed, only a single strepsirrhine. Strepsirrhines, which represented 31% of the dataset, exhibited a tendency for more seasonal breeding and for fewer females per group than did haplorrhines. Interestingly, however, the inclusion of a greater diversity of primate taxa in the analysis did not substantially alter the results, which agree with studies documenting a relationship between the number of males and the number of females in primate groups [10], [16], [18], [57], [58], [61].

### Female Spatial Distribution and Reproductive Monopolization

In accord with previous comparative studies [10], [16], [61], the best predictor of the number of males in a primate group was the spatial patchiness of females measured as the number of females in that group. Indeed, a previous study found that evolutionary changes in the number of males in a group lags behind the change in the number of females [61]. Thus, group composition can be a strong indicator of male monopolization potential, perhaps because resident males can have priority or exclusive mating with group-living females (e.g., Verreaux's sifaka, *Propithecus verreauxi* [92], [93]), and may be a key factor used by dispersing individuals as they make emigration decisions (e.g., olive baboons, *Papio anubis* [94]; ringtailed lemurs, *Lemur catta* [95]).

Spatial cohesion of group members did not influence the number of males. This finding was surprising given that dispersion can reduce a male's ability to exclude rivals [10], [12], [13]. Spatial monopolization of females may translate poorly into actual reproductive monopolization for several reasons. First, physical presence in a group does not ensure access to reproductive opportunities. Heymann [60] found a distinction was necessary between breeding and natal adult males in classifying the mating system of golden lion tamarins (*Leontopithecus rosalia*), and, in fact, the modal mating system type changed when natal males were excluded from consideration. In species exhibiting female philopatry, males often do not reproduce until they leave the natal social group [94], [96]–[99].

Second, physical presence in a group does not ensure the exclusion of rival males from reproduction. Males with inferior competitive abilities unable to use preferred reproductive strategies may utilize alternative reproductive tactics, such as acting as a satellite to an established social group, engaging in extra-group copulations with group females, or engaging in furtive copulations within the group [100]–[105]. In other cases, such as in species exhibiting influxes of males during female estrous periods, group composition may change during times when reproduction takes place [59], [102], [106]–[108]. Following Nunn [10], our study examined male number as the typical number of males present in a social group. Males also can enhance their reproductive success in ways that are not reflected in measures of male group size, such as through extra-group copulations (e.g., fat-tailed dwarf lemurs, *Cheirogaleus medius* [109]; Verreaux's sifaka, *Propithecus verreauxi* [110]).

Third, female mate choice and counterstrategies may explain the lack of fit between female spatial cohesion and the number of males in a group. For example, females may use the strategy of influencing group membership to reduce male monopolization potential (e.g., Verreaux's sifaka, *Propithecus verreauxi* [93]) rather than reducing spatial cohesion, especially when predation risk is high [111]. Female counterstrategies to monopolization may be behavioral, such as engaging in surreptitious matings with other males [100], [101], [103]–[105], or physiological, through mechanisms such as cryptic female choice [112], [113]. Indeed, estrous synchrony may be a female counterstrategy to oppose male monopolization attempts by increasing the number of males in a group [17]. Once males reside in a group, female mate choice affects reproductive skew in some mammalian species, including primates [67], [114]–[119]. Female choice can result in either high reproductive skew (squirrel monkeys, *Saimiri oerstedi* [114]; Barbary macaques *Macaca sylvanus* [115]) or low reproductive skew (woolly spider monkeys, *Brachyteles arachnoides* [118]; rhesus macaques, *Macaca mulatta* [119]), depending on whether females prefer to mate with one or many males.

Finally, our measure of spatial cohesion may have been too qualitative to capture variation in monopolization potential. In fission-fusion species, males may continue to maintain close proximity and to control female mating opportunities by staying with subgroups that include estrous females. Unfortunately, the data for a more refined analysis are not currently available. Nevertheless, we did find that species with dispersed social systems do not differ from species with cohesive social systems even though individuals in the dispersed systems forage alone. These results suggest that spatial cohesion is not a good predictor of the number of males in primate social groups even though males are theoretically better able to spatially monopolize females in cohesive and fission-fusion systems.

### Temporal Distribution of Reproductive Opportunities

The temporal distribution of reproductive opportunities also influenced male monopolization potential. All three measures of temporal distribution of females were generally related to the number of males in primate groups, although support varied depending on the measure we used. In particular, we found strongest support for an effect of expected female overlap, which might be expected because it is the most direct estimate of reproductive synchrony that we analyzed. When demographic and life history conditions favor estrous synchrony, adult sex ratios in primate groups become less skewed. The length of the birth season, used as a proxy for the breeding season, also showed a tendency to covary with the number of males independently of the number of females. In this case, a longer birth season favored fewer males in the group, as expected. Finally, greater breeding seasonality seemed to favor more males in primate groups. This latter estimate of reproductive synchrony was the weakest, with slightly less than 90% of the regression coefficients in the predicted positive direction. However, sample sizes were also the smallest for this variable, and thus the statistical power to detect effects was likely to be lowest among the three measures of temporal effect. Breeding season duration and breeding seasonality may be poorer measures of male monopolization potential because (i) reproductive seasonality does not necessarily translate into estrous synchrony (e.g., ringtailed lemurs, *Lemur catta* [120]; gray mouse lemurs, *Microcebus murinus* [121]), (ii) males may be able to monopolize females despite estrous synchrony when groups are small (e.g., cercopithecines [58]) or probabilistic signals of female fertility are present [122], and (iii) females are able to exercise mate choice (reviewed in [123], [124]).

Interestingly, the number of females in a group appears to incorporate some temporal effects in statistical models. Females may include temporal factors, such as the degree of reproductive synchrony, in their decisions about group membership. However, by using a multiple regression model, the effects of reproductive synchrony on the number of males were independent of the number of females.

### Conclusion

Previous studies of the causes and consequences of sociality focused mainly on group composition, while more recent studies have focused on mating and reproductive skew [27], [125], [126]. Sexual selection theory predicts that both group composition and reproductive skew reflect male monopolization potential. By broadening previous datasets to include a more diverse sample of primate species, we were able to conduct a more expansive test of the relationship between female distribution and male distribution. Our study found that the number of females in groups is the primary predictor of the distribution of male primates, with female reproductive synchrony playing a secondary, but still important, role. Thus, as originally posed by Emlen and Oring [1], both the spatial and temporal distribution of reproductive opportunities determines the ability of males to monopolize access to fertile females. While researchers often examine dispersal patterns (e.g., [55], [96], [97], [127], [128]), few studies focus on the strategies individuals use to influence group membership (but see [93]). The importance of group composition for male monopolization potential suggests that an important area of future research is the investigation of the strategies residents use to influence group membership.

## Supporting Information

Table S1 Data used in the Carnes et al. analysis of the effects of the distribution of female primates on the number of males
(DOC)
