# Supplementary material for: Effects of the Distribution of Female Primates on the Number of Males
Source: PLoS One. 2011 May 16;6(5):e19853. doi: 10.1371/journal.pone.0019853 (PMC3095636; doi:10.1371/journal.pone.0019853)
Supplement: Table S1 — Data used in the Carnes et al. analysis of the effects of the distribution of female primates on the number of males (DOC) [file pone.0019853.s001.doc]

| Genus and species | (A) Breeding season Duration | (B) Estrus Duration | (C) Number Females | (D) Number Males | (E) Expected overlap | (F) Dispersion | (G) 'r' seasonality value | Reference Exceptions |
| --- | --- | --- | --- | --- | --- | --- | --- | --- |
| *Alouatta caraya* | 365 | 2.9 | 2 | 1 | 0 | C | 0.32 |  |
| *Alouatta palliata* | 365 | 3 | 8 | 2 | 0.7 | C | 0.2 |  |
| *Alouatta seniculus* | 365 | 2.8 | 2.5 | 1.5 | 0.1 | C | 0.2 | F) Pope 1990 |
| *Aotus trivirganus* | 365 | --- | 1 | 1 | 0 | C | 0.34 | A)Harcourt 1995 |
| *Ateles paniscus* | 365 | 6 | 16 | 5 | 9.6 | F | 0.37 |  |
| *Avahi laniger* | 59 | --- | 1 | 1 | 0 | F | --- |  |
| *Brachyteles arachnoides* | 153 | 2 | 15 | 9.8 | 5.7 | C | 0.64 |  |
| *Callicebus moloch* | 212 | --- | 1 | 1 | 0 | C | 0.82 | A) Valeggia et al. 1999, C&D) Plavcan 2004, F) Robinson 1979 |
| *Callimico goeldii* | 91 | 7 | 2.7 | 1.3 | 6.4 | C | --- | B) van Schaik et al 1999, C&D) Heymann 2000, F) Porter 2001 |
| *Callithrix jacchus* | 365 | 28 | 2.9 | 2.7 | 6.3 | C | 0.2 | F) Digby 1999 |
| *Cebuella pygmaea* | 184 | 6 | 1.4 | 1.4 | 0 | C | 0.15 | B) van Schaik et al 1999, C&D) Heymann 2000 |
| *Cebus apella* | 92 | 5 | 2.3 | 2 | 1.2 | C | 0.66 | F) Stevenson 1998 |
| *Cebus capucinus* | 212 | 5.5 | 4 | 5.5 | 1.5 | C | 0.46 | F) Boinski & Campbell 1996 |
| *Cebus olivaceus* | 124 | 5.5 | 6 | 1 | 9.3 | C | 0.66 | F) Robinson 1988 |
| *Cercopithecus ascanius* | 182 | 3 | 9.5 | 1 | 4.1 | C | 0.25 | F) Cords 1987 |
| *Cercopithecus mitis* | 120 | 2 | 18 | 1 | 12 | C | 0.73 | F) Cords 1987 |
| *Cheirogaleus medius* | 61 | 2 | 1 | 1 | 0 | D | --- |  |
| *Chlorocebus aethiops* | 92 | 33 | 4.3 | 3 | 92 | C | 0.85 | F) Melnick & Pearl 1987 |
| *Colobus guereza* | 365 | 8 | 3 | 1 | 0.6 | C | --- | B) Harris & Monfort 2006, F) Oates 1977 |
| *Daubentonia madagascariensis* | 365 | 8 | 1 | 1 | 0 | D | --- |  |
| *Erythrocebus patas* | 62 | 2 | 13 | 3 | 20 | C | 0.85 | F) Cords 1987 |
| *Eulemur fulvus rufus* | 28 | 2.5 | 2.8 | 3.8 | 8.4 | C | 1 | A) Ostner et al 2008, C&D) Ostner & Kappeler 2004 |
| *Eulemur macaco flavifrons* | 22 | 1.5 | 3.1 | 4 | 5.1 | C | --- |  |
| *Eulemur macaco macaco* | 22 | 1.5 | 3.5 | 3.2 | 9.2 | F | --- | A) Simmen 2007 |
| *Eulemur mongoz* | 61 | 1 | 1 | 1 | 0 | C | 0.99 |  |
| *Eulemur rubriventer* | 61 | --- | 1 | 1 | 0 | C | 0.92 |  |
| *Galago moholi* | 120 | 2 | 8.1 | 16 | 2.7 | D | 0.43 |  |
| *Gorilla beringei beringei* | 365 | 1.5 | 7 | 2 | 0.1 | C | --- | A) Watts 1998, B) Robbins 2001, C&D) Robbins 1999, F) Watts 2000 |
| *Gorilla gorilla gorilla* | 365 | 3 | 4.4 | 1.8 | 0.2 | C | 0.05 | F) Robbins 2004 |
| *Hapalemur griseus* | 123 | 1 | 1.3 | 1.3 | 0 | C | 0.88 | A) Mittermeier et al 2006, C&D) Nievergelt 2002 |
| *Hylobates lar* | 122 | 4 | 1 | 1 | 0 | C | --- | A) Savini et al. 2008, B) van Schaik et al. 1999, F) Leighton 1987 |
| *Indri indri* | 31 | --- | 1 | 1 | 0 | C | --- |  |
| *Lagothrix lagotricha* | 184 | 3.1 | 9.8 | 3.3 | 4.3 | C | 0.59 | A) Nishimura 2003, C&D) Plavcan 2004, F) Stevenson 1998 |
| *Lemur catta* | 38 | 1 | 4 | 4.5 | 1.5 | C | 0.97 |  |
| *Leontopithecus rosalia* | 212 | 18 | 1.5 | 2 | 2.9 | C | 0.68 | A) Miller et al. 2006, B) van Schaik et al. 1999, C&D) Heymann 2000, F) Dietz and Baker 1993 |
| *Lophocebus albigena* | 212 | 4 | 7 | 5 | 2.6 | C | --- | F) Melnick & Pearl 1987 |
| *Loris tardigradus* | 365 | 2 | 2.5 | 1 | 0 | D | --- | C&D) Radhakrisna & Singh 2002 |
| *Macaca fascicularis* | 123 | 15 | 6.8 | 4 | 54 | C | 0.61 |  |
| *Macaca fuscata* | 46 | 11 | 9 | 3 | 97 | C | 0.92 |  |
| *Macaca mulatta* | 82 | 9 | 9 | 2.5 | 62 | C | --- |  |
| *Macaca nemestrina* | 365 | 13 | 22 | 3 | 47 | C | 0.25 |  |
| *Macaca radiata* | 92 | 5 | 9 | 7 | 26 | C | 0.86 |  |
| *Macaca silenus* | 365 | 18 | 7 | 1.8 | 15 | C | 0.33 |  |
| *Macaca sinica* | 66 | 14 | 9.5 | 5 | 97 | C | 0.61 |  |
| *Macaca sylvanus* | 76 | 14 | 11 | 9 | 95 | C | 0.92 |  |
| *Microcebus murinus* | 28 | 0.1 | 7 | 4 | 0.2 | D | --- | C&D) Radspiel 2000 |
| *Miopithecus talapoin* | 59 | 11 | 27 | 13 | 100 | C | --- |  |
| *Nycticebus coucang* | 365 | 3.5 | 1 | 1 | 0 | D | --- |  |
| *Otolemur crassicaudatus* | 61 | 14 | 1 | 1 | 0 | D | --- |  |
| *Otolemur garnetti* | 61 | 5.8 | 1 | 1 | 0 | D | --- |  |
| *Pan paniscus* | 365 | 15 | 8 | 8 | 14 | F | --- | F) Nishida & Hiraiwa-Hasegawa 1987 |
| *Pan troglodytes* | 365 | 14 | 35 | 10 | 76 | F | 0.09 | F) Nishida & Hiraiwa-Hasegawa 1987 |
| *Papio anubis* | 365 | 6 | 34 | 14 | 31 | C | 0.19 | F) Melnick & Pearl 1987 |
| *Papio cynocephalus* | 365 | 9 | 13 | 8 | 13 | C | 0.14 | F) Melnick & Pearl 1987 |
| *Papio hamadryas* | 365 | 5 | 2 | 1 | 0.1 | F | --- |  |
| *Papio ursinus* | 365 | 9 | 15 | 7 | 17 | C | 0.42 | F) Melnick & Pearl 1987 |
| *Perodicticus potto* | 184 | 2 | 1 | 1 | 0 | D | 0.77 | A) Fitch et al. 2003, B) van Schaik et al. 1999 |
| *Phaner furcifer* | 15 | --- | 1 | 1 | 0 | D | --- |  |
| *Piliocolobus badius* | 244 | 5 | 16 | 8 | 14 | C | 0.37 | B) van Schaik et al. 1999, F) Struhsaker 2000 |
| *Pongo pygmeaus* | 365 | 31 | 16 | 9 | 79 | F | --- | C, D, F) Singleton & van Schaik 2001 |
| *Propithecus edwardsi* | 61 | 2 | 1.4 | 1.5 | 0 | C | --- | A,C,D) Pochron 2005 |
| *Propithecus verreauxi verreauxi* | 90 | 1.5 | 2.5 | 2.5 | 0.3 | C | --- | C&D) Brockman 1994 |
| *Saguinus fuscicollis* | 151 | 10 | 1 | 1.9 | 0 | C | 0.63 | F) Goldizen et al. 1996 |
| *Saguinus oedipus* | 122 | 11 | 1 | 2 | 0 | C |  | F) Savage 1996 |
| *Saimiri oerstedi* | 59 | 2 | 16 | 10 | 30 | C | 0.94 | F) Boinski 1999 |
| *Saimiri sciureus* | 61 | 2 | 23 | 7 | 45 | C | --- |  |
| *Semnopithecus entellus* | 365 | 4 | 19 | 6 | 6.4 | C | --- | F) Newton 1987 |
| *Tarsius bancanus* | 90 | 3 | 1 | 1 | 0 | D | --- |  |
| *Tarsius syrichta* | 122 | 4.5 | 1 | 1 | 0 | D | --- |  |
| *Theropithecus gelada* | 365 | 9 | 4 | 1 | 1.4 | F | 0.09 | F) Fedigan 1992 |
| *Varecia variegata rubra* | 61 | 0.4 | 2 | 1 | 0 | C | --- | A,C,D) Vasey 2007 |

Unless otherwise noted, data were drawn from the following sources: (Mitani et al. 1996; Nunn & Barton 2000; Janson and Verdolin 2005; Treatman-Clark 2006; Campbell et al. 2010). Expected overlap values were calculated following the procedure outlined in the methods section. Dashed lines indicate data were not available.

References

Boinski S (1999) The social organizations of squirrel monkeys: Implications for ecological models of social evolution. Evol Anthropol 8: 101-112.

Boinski S, Campbell AF (1996) The huh vocalization of white-faced capuchins: A spacing call disguised as a food call? Ethology 102: 826-840.

Brockman DK (1994) Reproduction and mating system of Verreaux’s sifaka, *Propithecus verreauxi verreauxi*, at Beza Mahafaly, Madagascar, Ph.D. dissertation, Yale University, New Haven, CT.

Campbell CJ, Fuentes A, MacKinnon KC, Bearder SK, Stumpf RM (2010) Primates in Perspective. New York: Oxford University Press. 864 p.

Cords M (1987) Forest guenons and patas monkeys: male-male competition in one male groups. In: Smuts BB, Cheney DL, Seyfarth RM, Wrangham RW, Struhsaker TT, editors. Primate societies. Chicago: The University of Chicago Press. pp 98-111.

Dietz JM (1993) Polygyny and female reproductive success in golden lion tamarins, *Leontopithecus rosalia*. Anim Behav 46: 1067-1078.

Digby LJ (1999) Sexual behavior and extragroup copulations in a wild population of common marmosets (*Callithrix jacchus*). Folia Primatol 70: 136-145

Fedigan LM (1992) Primate paradigms: Sex roles and social bonds. Chicago: University of Chicago Press. 424 p.

Fitch-Snyder H, Jurke M (2003) Reproductive patterns in pygmy lorises (*Nycticebus pygmaeus*): Behavioral and physiological correlates of gonadal activity Zoo Biol 22: 15-32.

Goldizen AW, Mendelson J, van Vlaardingen M, Terborgh J (1996) Saddle-back tamarin (*Saguinus fuscicollis*) reproductive strategies: Evidence from a thirteen-year study of a marked population. Am J Primatol 38: 57-83.

Harris T, Monfort SL (2006) Mating behavior and endocrine profiles of wild black and white Colobus monkeys (*Colobus guereza*): Toward an understanding of their life history and mating system. Am J Primatol 68: 383-396.

Heymann EW (2000) The number of adult males in callitrichine groups and its implications for callitrichine social evolution. In: Kappeler PM, editor. Primate males. Cambridge: Cambridge University Press. pp 64-71.

Janson C, Verdolin J (2005) Seasonality of primate births in relation to climate. In: Brockman DK, van Schaik CP, editors. Seasonality in primates: Studies of living and extinct human and non-human primates. Cambridge: Cambridge University Press. pp 307–350.

Korstjens AH, Noë R (2004) Mating system of an exceptional primate, the olive colobus (*Procolobus verus*). Am J Primatol 62: 261-273.

Leighton DR (1987) Gibbons: Territoriality and monogamy. In: Smuts BB, Cheney DL, Seyfarth RM, Wrangham RW, Struhsaker TT, editors. Primate societies. Chicago: The University of Chicago Press. pp 135-145.

Melnick DJ, Pearl MC (1987) Cercopithecines in mulitmale groups: Genetic diversity and population structure. In: Smuts BB, Cheney DL, Seyfarth RM, Wrangham RW, Struhsaker TT, editors. Primate societies. Chicago: The University of Chicago Press. pp 121-134.

Miller KE, Bales KL, Ramos JH, Dietz JM (2006) Energy intake, energy expenditure, and reproductive costs of female wild golden lion tamarins (*Leontopithecus rosalia*). Am J Primatol 68: 1037-1053.

Mitani JC, Gros-Louis J, Manson JH (1996) Number of males in primate groups: Comparative tests of competing hypotheses. Am J Primatol 38: 315-332.

Mittermeier RA, Tattersall I, Konstant WR, Meyers DM, Mast RB (1996) Lemurs of madagascar. Conservation International, Washington, DC. 520 p.

Newton PN (1987) The social organization of forest Hanuman langurs (*Presbytis entellus*). Int J Primatol 8: 199-232.

Nievergelt, CM, Mutschler T, Feistner ATC, Woodruff DS (2002) Social system of the alaotran gentle lemur (*Hapalemur griseus alaotrensis*): Genetic characterization of group composition and mating system. Am J Primatol 57: 157-176.

Nishida T, Hiraiwa-Hasegawa M (1987) Chimpanzees and bonobos: Cooperative relationships among males. In: Smuts BB, Cheney DL, Seyfarth RM, Wrangham RW, Struhsaker TT, editors. Primate societies. Chicago: The University of Chicago Press. pp 165-178.

Nishimura A (2003) Reproductive parameters of wild female *Lagothrix lagotricha*. Int J Primatol 24: 707-722.

Nunn CL, Barton RA (2000) Allometric slopes and independent contrasts: A comparative test of Kleiber's law in primate ranging patterns. Am Nat 156: 519-533.

Oates JF (1977) Social life of a black and white colobus monkey, *Colobus guereza*. Zeit Fur Tierpsych 45: 1-60.

— (1994) The natural history of African colobines. In: Davies AG, Oates JF, editors. Colobine monkeys. Cambridge: Cambridge University Press. pp 75-128.

Ostner J, Kappeler P, Heistermann M (2008) Androgen and glucocorticoid levels reflect seasonally occurring social challenges in male redfronted lemurs (*Eulemur fulvus rufus*). Behav Ecol Sociobiol 62: 627-638.

Plavcan JM (2004) Sexual selection, measures of sexual selection, and sexual dimorphism in primates. In: Kappeler P van Schaik CP, editors. Sexual selection in primates. Cambridge: Cambridge University Press. pp 230-252.

Pochron ST, Wright PC (2005) Testes size and body weight in the Milne-Edwards' sifaka (*Propithecus edwardsi*) of Ranomafana National Park, Madagascar, relative to other strepsirhine primates. Folia Primatol 76: 37-41.

Pope TR (1990) The reproductive consequences of male cooperation in the red howler monkey: paternity exclusion in multi-male and single-male troops using genetic markers. Behav Ecol Sociobiol 27: 439-446.

Porter LM (2001) Social organization, reproduction and rearing strategies of *Callimico goeldii*: New clues from the wild. Folia Primatologica 72: 69-79.

Radespiel U (2000) Sociality in the gray mouse lemur (*Microcebus murinus*) in northwestern Madagascar. Am J Primatol 51: 21-40.

Radhakrishna S, Singh M (2002) Home range and ranging pattern in the slender loris (*Loris tardigradus lydekkerianus*). Primates 43: 237-248.

Richard AF (1978) Behavioral variation: Case study of a Malagasy lemur. Lewisburg: Bucknell University Press. 213 p.

Robbins M (2001) Variation in the social system of mountain gorillas: The male perspective. In: Robbins MM, Sicotte P, Stewart KJ, editors. Mountain gorillas: Three decades of research at Karisoke. Cambridge: Cambridge University Press. pp 29-58.

Robbins MM (1999) Male mating patterns in wild multimale mountain gorilla groups. Anim Behav 57: 1013-1020.

Robbins MM, Bermejo M, Cipolletta C, Magliocca F, Parnell RJ, et al. (2004) Social structure and life-history patterns in western gorillas (*Gorilla gorilla gorilla*). Am J Primatol 64: 145-159.

Robinson JG (1979) Vocal regulation of use of space by groups of titi monkeys *Callicebus moloch*. Behav Ecol Sociobiol 5: 1-15.

— (1988) Group-size in wedge-capped capuchin monkeys *Cebus olivaceus* and the reproductive success of males and females. Behav Ecol Sociobiol 23: 187-197.

Savage A, Giraldo KH, Soto KH, Snowdon CT (1996) Demography, group composition, and dispersal in wild cotton-top tamarin (*Saguinus oedipus*) groups. Am J Primatol 38 :85-100.

Savini T, Boesch C, Reichard UH (2008) Home-range characteristics and the influence of seasonality on female reproduction in white-handed gibbons (*Hylobates lar*) at Khao Yai National Park, Thailand. Am J Phys Anthropol 135: 1-12.

Simmen B, Bayart F, Marez A, Hladik A (2007) Diet, nutritional ecology, and birth season of *Eulemur macaco* in an anthropogenic forest in Madagascar. Int J Primatol 28: 1253-1266.

Singleton I, van Schaik CP (2001) Orangutan home range size and its determinants in a Sumatran swamp forest. Int J Primatol 22: 877-911.

Stevenson PR (1998) Proximal spacing between individuals in a group of woolly monkeys (*Lagothrix lagotricha*) in Tinigua National Park, Colombia. Int J Primatol 19: 299-311.

Stevenson PR, Quinones MJ, Ahumada JA (1998) Effects of fruit patch availability on feeding subgroup size and spacing patterns in four primate species at Tinigua National Park, Colombia. Int J Primatol 19: 313-324.

Struhsaker TT (2000) Variation in adult sex ratios of red colobus monkey social groups: implications for interspecific comparisons. In: Kappeler PM, editor. Primate males. Cambridge: Cambridge University Press. pp 108-119.

Valeggia CR, Mendoza SP, Fernandez-Duque E, Mason WA, Lasley B (1999) Reproductive biology of female titi monkeys (*Callicebus moloch*) in captivity. Am J Primatol 47: 183-195.

van Schaik CP, van Noordwijk MA, Nunn CL (1999) Sex and social evolution in primates. In: Lee PC, editor. Comparative primate socioecology. Cambridge: Cambridge University Press. pp 204-240.

Vasey N (2007) The breeding system of wild red ruffed lemurs (*Varecia rubra*): a preliminary report. Primates 48: 41-54.

Watts DP (1998) Seasonality in the ecology and life histories of mountain gorillas (*Gorilla gorilla beringei*). Int J Primatol 19: 929-948.

— (2000) Causes and consequences of variation in male mountain gorilla life histories and group membership. In: Kappeler PM, editor. Primate males. Cambridge: Cambridge University Press. pp 169-180.
